# A case of successful chemotherapy in an elderly double‐hit lymphoma patient with a giant tumor, severe renal impairment, and unfavorable performance status

**DOI:** 10.1111/ggi.14823

**Published:** 2024-02-08

**Authors:** Ryo Yamamoto, Raita Fukasawa, Shuntaro Serisawa, Naoto Takenoshita, Yoshitsugu Kaneko, Yusuke Ogawa, Tomohiko Sato, Hidekazu Kanetaka, Soichiro Shimizu

**Affiliations:** ^1^ Department of Comprehensive Geriatric Medicine Tokyo Medical University Hospital Tokyo Japan

Dear Editor,

Double‐hit lymphoma (DHL) is associated with *MYC* gene translocation and either the *BCL2* or *BCL6* gene or both translocations, and is considered a subtype of diffuse large B‐cell lymphoma (DLBCL) among the malignant lymphomas. We encountered a case of DHL in an elderly patient with a giant tumor, severe renal dysfunction, and unfavorable performance status (PS).

The patient was a 75‐year‐old woman. Three months previously, she had experienced chest pain. A computed tomography (CT) examination displayed an anterior mediastinal mass and an intrahepatic mass. Two weeks after, she was brought to our hospital by ambulance owing to dyspnea, and was admitted. B‐cell malignant lymphoma was suspected on thyroid biopsy.

A physical examination on her admission showed that she had E3V4M5‐level disturbance of consciousness in the Glasgow Coma Scale, epigastric tenderness, and pitting edema all over her body. The results of her blood test on admission are shown in Fig. 1A. Head magnetic resonance imaging (MRI) revealed no intracranial metastasis. A positron emission tomography‐CT (PET‐CT) scan taken before admission displayed a giant tumor with signal accumulation in the anterior mediastinum and liver, and lesions with signal accumulation in the parotid gland, pancreas, adrenal glands, and systemic lymph nodes (Fig. 1B).

**Figure 1:**
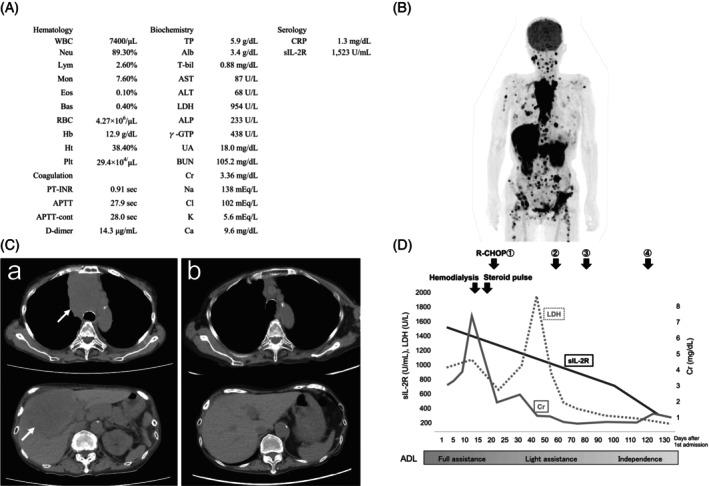
Blood examination, PET‐CT and CT imaging, and progress of the patient. (A) Blood examination findings on admission. (B) PET‐CT scan taken before admission. Signal accumulation was consistent with the presence of a giant tumor in the liver and in the anterior mediastinum that was connected to the thyroid gland. In addition, signal accumulation was observed in the neck, armpits, lung, kidney, adrenal glands, intraperitoneal cavity, groin, and thighs. (C) Comparison of CT images taken before and after chemotherapy. (a) Before chemotherapy. The anterior mediastinal lesion (top) and intrahepatic lesions (bottom) are indicated by white arrows. (b) After four courses of R‐CHOP therapy. A clear improvement was observed in both the anterior mediastinal lesion (top) and the intrahepatic lesion (bottom). (D) Progress of the patient. After steroid pulse therapy, her renal function improved, and she was taken off hemodialysis. Subsequently, she underwent a total of four courses of R‐CHOP therapy: sIL‐2R improved to the baseline level, and ADL improved to independence. LDH showed a slight decrease after steroid pulse therapy, and a temporary increase due to tumor destruction after the first R‐CHOP therapy, but a downward trend was observed thereafter. Alb, albmin; ALP, alkaline phosphatase; ALT, alanine aminotransferase; APTT, activated partial thromboplastin time; AST, asparate aminotransferase; Bas, basophil; BUN, blood urea nitrogen; Ca, calcium; Cl, chlorine; Cr, creatinine; CRP, c‐reactive protein; Eos, eosinophil; Hb, hemoglobin; Ht, hematocrit; K, kalium; LDH, lactate dehydrogenase; Lym, lymphocyte; Mon, monocyte; Na, natrium; Neu, neutrophil; Plt, platelet; PT‐INR, International normalized ratio of prothorombin time; RBC, red blood cell; sIL‐2R, soluble interleukin‐2 receptor; T‐bil, total bilirubin; TP, total protein; γ‐GTP, γ‐glutamyl trans peptidase; UA, uric acid; WBC, white blood cell.

Considering the patient's condition, especially considering the risk of tumor lysis syndrome, treatment of her malignant lymphoma by chemotherapy was concluded to be difficult.

The patient's renal dysfunction temporarily increased to about Cr 7.0 mg/dL, and hemodialysis was started on the 11th hospital day. On the 12th hospital day, the patient was treated with steroid pulse therapy with methylprednisolone 1000 mg/day for 3 days. Her renal function subsequently improved, and she was weaned off dialysis. Although there was no change in tumor burden, a decrease in lactate dehydrogenase (LDH) was observed. We were considering a best supportive care policy until we started administering steroids, but her general condition also improved, so we decided to try chemotherapy.

As the interim pathological results indicated that the patient had B‐cell lymphoma, rituximab was administered for the first time on day 22, followed by cyclophosphamide, doxorubicin hydrochloride, vincristine, and prednisolone (CHOP) therapy at two‐thirds of the reduced dose, which resulted in tumor shrinkage. The final report of the pathological examination was high‐grade B‐cell lymphoma with *MYC/BCL6* rearrangements; that is, DHL involving *MYC* and *BCL6*.

A total of four courses of rituximab‐cyclophosphamide, doxorubicin hydrochloride, vincristine, and prednisolone (R‐CHOP) therapy were then administered. Imaging results indicated further improvement (Fig. 1C), and her activities of daily living (ADL) also improved to almost independence. Figure 1D shows the patient's progress. However, the patient's condition subsequently worsened owing to tumor recurrence, and chemotherapy could not be continued. She survived for 12 months after onset of symptoms.

DHL involves translocation of the *MYC* gene, and translocation of either the *BCL2* or the *BCL6* gene or both, with *MYC*‐*BCL2* DHL in 2% to 8% of DLBCL patients and *MYC*‐*BCL6* DHL, which is a rare subtype, in 0.8% to 1% of patients. R‐CHOP therapy is considered to be ineffective for DHL,^1^ and no treatment has been established to date. Rituximab‐etoposide, prednisolone, oncovin, cyclophosphamide, and doxorubicin hydrochloride (R‐EPOCH), etc., are being considered as potential treatments.^2^ The median overall survival of *MYC*‐*BCL6* DHL patients is reported to be 17.2 months, and the 1‐year survival rate is 58%, which is considered to be similar to that of *MYC*‐*BCL2* DHL patients.^3^

One of the reasons that we performed chemotherapy on this patient was the improvement of her general condition and renal dysfunction after steroid pulse therapy. As her renal dysfunction was caused by interstitial nephritis, steroid administration may have resulted in its improvement. In addition, it is assumed that the patient's malignant lymphoma originally metastasized to the adrenal glands, resulting in a decrease in physiological steroid production, and steroid supplementation may have improved her general condition.

Another reason that chemotherapy was performed on this patient is associated with the cancer pharmacotherapy guidelines for the elderly.^4^ The guidelines suggest that comprehensive geriatric assessment (CGA) should not be used to determine treatment strategies for elderly DLBCL patients. As DLBCL often responds favorably to chemotherapy, we suggest that chemotherapy should be performed regardless of CGA results. In the present case, the possibility of DLBCL was considered in the pathological interim report, which encouraged us to try chemotherapy without relying on the CGA evaluation. However, the use of chemotherapy for DLBCL patient in these guidelines is only a “suggestion,” and as this patient was ultimately diagnosed as having DHL, we believe that more caution should be exercised in making decisions to perform chemotherapy.

Elderly patients with malignant lymphoma require a more comprehensive evaluation than younger patients regarding whether or not treatment should be performed. On the other hand, active treatment that is not necessarily bound by the results of geriatric functional evaluation may be effective, and we believe that this case demonstrates this point.

## Disclosure statement

The authors have no financial disclosures to declare.

## Data Availability

The data that support the findings of this study are available on request from the corresponding author. The data are not publicly available due to privacy or ethical restrictions.
